# Reading and Equity in Teacher Education: An Exploratory Study

**DOI:** 10.1177/1086296X251401121

**Published:** 2025-12-03

**Authors:** Rachel Heydon, Lori McKee, Elizabeth Akiwenzie, Emma Cooper, Bronwyn Johns, Pamela J. McKenzie, Marianne McTavish, Sandra Poczobut, Carla Ruthes Coelho, Melody Viczko, Zheng Zhang

**Affiliations:** 1 Faculty of Education, 6221 Western University, London, ON, Canada; 2 7235 College of Education, University of Saskatchewan, Saskatoon, SK, Canada; 3 Ojibway and Oneida Cultural Educator and Healer; 4 2152 Faculty of Social Science, University of Stirling, Stirling, UK; 5 Faculty of Information and Media Studies, Western University, London, ON, Canada; 6 8166 Language and Literacy Education Department, University of British Columbia, Vancouver, BC, Canada; 7 1270 Faculty of Education, St. Francis Xavier University, Antigonish, NS, Canada

**Keywords:** academic language, critical literacies, semiotics, higher education

## Abstract

Reading is central to higher education, but requires increased pedagogical and research attention. Practice and knowledge gaps have created threats to equity, though the precise nature of the reading/equity nexus in higher education is unknown. The Reading Pedagogies of Equity Project, a professional learning program and study designed with teacher educators, sought to produce insights into equity and reading in higher education. Oriented through critical posthumanism and a speculative pedagogies of qualitative inquiry methodology, the research team generated data with nine teacher educator participants. Data sources included the program pedagogies, discussions, and artifacts, plus pre- and post-program interviews. Data were analyzed through a thinking with theory approach focused on entanglement, diffractive reading, and enacted agency. The study identified textual, contextual, pedagogical, and readerly (im)materialities in academic reading that (dis)able opportunities for equity and the processes for producing this knowledge. Findings are significant for educators wanting to promote equity in/through reading.

This article reports on the Reading Pedagogies of Equity Project (RPEP) that provided, through professional learning for teacher educators, an interrogation of the concepts of reading and equity and their relationship within higher education. Reading in the literacy literature has been conceptualized in myriad ways, with posthuman orientations describing it as a practice that entangles human, social, and physical (im)materialities (Burnett et al., 2014). These entanglements (Barad, 2007) exert a form of enacted agency (Wargo, 2018) that produces effects, such as textual meanings. Relative to reading in higher education, entanglements might include (im)materialities such as lists of required readings that form the core of syllabi, students’ prior knowledge, and the demands of academic texts. Students’ perceived reading comprehension in these entanglements can affect their academic achievement (Desa et al., 2020), how they feel about themselves (Niccolini, 2019), and their involvement in the disciplinary domain of a course (Mazawi & Stack, 2020). Despite its importance and complexities, however, reading in higher education has been overshadowed in the literature by academic writing and treated predominantly as an instrumental skill (Baker et al., 2019). Consequently, a truncated view of reading in higher education proliferates, and there is more to be learned about reading and its connections to equity.

RPEP asked what could be learned about the reading/equity nexus in higher education if teacher educators participated in professional learning to explore reading within their own teaching. It recognized that higher education is a context “characterised by … alienation, exclusion and marginalisation” (Postma, 2016, p. 310) that calls for equity as a “grounded, localized understanding of situated ethics” (Pennycook, 2017, p. 30). Equity here is the outcome of entangled relations that generate complexity, diversity, and possibilities for all constituents of the planet to live well together (Murris, 2016).

RPEP pursued its query through a speculative pedagogies of qualitative research (Kuby & Christ, 2020) methodology, in which pedagogies are recognized as a form of research leading to new understandings of phenomena. RPEP selected the context of teacher education to learn with and about reading and equity as teacher education is fundamentally obliged to sow equitable pedagogical practices among teacher candidates while modeling such practices in academic reading engagements. Highlighting one cycle of RPEP professional learning, this article shares who and what became entangled during the program and how these entanglements enacted agency that produced lessons about reading and equity. The findings may hold significance for teacher education, making it more aware of what reading is and what it produces in everyday teacher education classrooms. They may also be significant for the advancement of professional learning in critical reading practices across domains.

## Orientation

RPEP was structured through an interrelated professional learning program and study oriented through critical posthumanism. Intrinsic to critical posthumanism is an ethico-onto-epistemology (Barad, 2007), a recognition of the inseparability between ethical values, seeing, knowing, doing, and becoming. Two of critical posthumanism's main assumptions directed RPEP's ethico-onto-epistemology. One, critical thinking is urgently needed to address the challenges of the current age. Braidotti (2019) argued that the earth has entered the epoch of the Anthropocene, a “multi-layered posthuman predicament” caused by humans’ devastating impacts on the planet, which includes “environmental, socioeconomic, and affect and psychic dimensions” (p. 32). This predicament foregrounds the relationships between humans and nonhumans and the damage that comes from “hierarchies based around a dominant notion of what humanity is, or should be” (Sidebottom, 2024). Two, critical thinking can respond to the Anthropocene by identifying “the power relations operational in and immanent to the production and circulation of knowledge” (Braidotti, 2019, p. 32). As a form of critical thinking, critical reading is a meticulous consideration of power and knowledge, their articulations and effects, and the relations that may help to produce “joyful or affirmative values and projects” (Braidotti, 2019, p. 33). We hoped that considering critical reading within and through RPEP could yield situated accounts and understandings of reading and equity in teacher education that could be used as resources for new relations in and of reading.

Within critical posthumanism, reading is a complex practice that involves “making sense of how others [signify] ideas using a wide range of text forms” and “drawing from knowledge and experience within sociocultural and material contexts” (Stagg Peterson et al., 2020, p. 1). Reading includes the cocreation of meaning through the intra-relation (Barad, 2007) of situated (im)materialities. Burnett et al. (2014) coined the term “(im)materialities” to refer to the range of “space, mediation, embodiment and stuff” (p. 100), the sociomaterial entanglements of the moment-by-moment doing of literacies (Leander & Boldt, 2012). Thus, reading is never solely an individual cognitive accomplishment; it is the effect of much (Burnett et al., 2014), such as people, texts, and places. And because this orientation is interested in reading diffractively—that is, in ways that attend to the differences in and productions of relational encounters (Lenz Taguchi, 2012), including by reading beyond binaries (Braidotti, 2019)—RPEP invited the consideration of academic reading with a diversity of research (e.g., multimodal, multilingual, multidiscursive, and decolonial).

The relational ontology of posthumanism decenters the human in reading. Reading is not all in the (human) head as people work with texts, but texts also work on people, affecting how they think, feel, and may participate in the world (Heydon et al., 2021). This decentering highlights the entanglements and movements of the time/space/matter in which readers are implicated (Kuby & Rucker, 2016), allowing for a (re)consideration of reading in teacher education. Tangibly, RPEP's ethico-onto-epistemology oriented all of its professional learning and research activities, including its insistence on thinking with materials (e.g., texts), documenting what was therein produced through this thinking, and sharing this thinking with others. An RPEP atelierista (i.e., an artist pedagogue) facilitated this thinking with/through modes (Kind, 2013) by, for example, representing and sharing back to participants in the professional learning ideas from the program's texts and discussions.

Further to RPEP's orientation is the concept that no knowledge, including knowledge of reading pedagogies, is an autonomous (Street, 1984) product that can be passed from person to person. Knowledge is ideological (Street, 1984), “grounded in how it is used, and how it relates to power” (Pahl & Rowsell, 2005, p. 14). The relationality of knowledge has curricular implications. A knowledge-as-product model minoritizes certain knowledge and limits collectively created knowledge (e.g., Freire, 1970). Reading in education has traditionally involved imposing hegemonic knowledge (Apple, 2004). In contrast, RPEP sought to generate knowledge *with* teacher educators and texts, inviting participants to think with the location of reading pedagogies. Given that RPEP was held in the settler colonial nation state called Canada, location here meant reckoning with the implications of settler colonialism and that we are all, on Turtle Island, treaty people (Chambers, 2012). Co-author, Elizabeth Akiwenzie, an Indigenous cultural educator and healer, was a key figure in RPEP in promoting thinking with place.

## Literature

We found in our literature review of reading and higher education that a posthuman project such as RPEP could make a contribution to the conversation on reading, equity, and teacher education. Our review found that skills-based approaches to reading have been prioritized in the research, with recent, important critiques coming from studies informed by sociocultural theory. This literature has suggested some of the (im)materialities involved in reading in higher education and what their effects may be, especially on students and their academic performance. Our synthesis of extant research suggests these (im)materialities are contextual (i.e., concerning the context of higher education), textual (i.e., concerning the text), pedagogical (i.e., concerning teaching and learning circumstances), and/or readerly (i.e., concerning the reader). To be added to this conversation are sociomaterial considerations of reading that decenter the reader to provide more situated explorations of what an ethics of equity might be in and through reading. The literature on teacher education and professional learning for teacher educators proposes teacher education as a prime locale for the development of such contributions.

### Reading in Higher Education

Reading has been seen across the literature as a requirement for successful participation in higher education (Desa et al., 2020). Despite its importance, a scoping study by Baker et al. (2019) found that contextually, higher education has focused on academic writing with scant consideration of reading outside of skills-based approaches, rendering the complexities of reading invisible. Attempts to make these complexities visible have centered on readers and strategies to assist them. Mason and Warmington (2024), for instance, studied higher education students’ experiences with academic reading. They found that students undertake this reading as a grudging act, described as “a struggle,” “a chore,” and “pointless” (p. 840), and recommended instructional strategies to mitigate this negative disposition. Similar in focus, Andrianatos (2019) studied faculty's and students’ perceptions “about the variables of the reading process” (p. 1). Andrianatos found “barriers” concerning the “reader” (p. 4), “text” (p. 5), “task,” and “socio-cultural context” (p. 6) that “hamper students’ reading development” (p. 7). These multipronged barriers provide an entrée to considering myriad (im)materialities.

The invisibility of reading beyond a skill has also been discussed in the sociocultural literature as creating particular barriers for particular students. Namely, it has been noted as threatening to minoritize the knowledge of students who come from outside the discourse community of a text; for example, Baker et al. (2019) found students “from non-dominant language backgrounds” to be vulnerable to minoritization “due to unfamiliarity with the tacit assumptions and hidden cultural values associated with disciplinary language and privileged forms of textual practice” (p. 145). The obfuscation of the demands and complexities of reading, they contended, can create linguistic and epistemological barriers to academic success. These barriers have been “often misinterpreted as a lack of capacity or motivation for succeeding in higher education,” hence “fueling deficit models” (p. 145) of minoritized students.

When considering academic reading beyond a focus on the student, diverse literatures suggest there may be other reading entanglements that affect equity in higher education. These could be those related to citational practices (Anderson & Christen, 2019), the ways in which course readings construct scholarly domains with insiders and outsiders (Maringe & Sing, 2014), and limitations in institutional knowledge of equity (McNair et al., 2020), decolonization (Indspire, 2018), and critical reading (Sutherland & Incera, 2021). These suggestions provided lines of inquiry for RPEP when it decentered the student.

### Teacher Education

The literature proposes that teacher education is a promising setting for querying reading and equity. Teacher education has long been regarded as an opportunity for promoting equity, although equity-focused scholars, such as those from Project RITE (Rethinking Initial Teacher Education for Equity; Cochran-Smith et al., 2016), have asserted that the concept can be murky. Following analyses of conceptions of equity, Project RITE recognized it as “primarily a commitment to justice and fairness based on the assumption that in many countries … educational opportunities, resources, and outcomes are unequally and unfairly distributed among groups differentiated by race/ethnicity/language, socioeconomic status, gender, and disability” (Cochran-Smith et al., 2016, p. 69). Teacher education might forward equity, according to literature in this vein, by “disrupting the default modes of schooling” that have (re)created social inequities (Johnston & Hayes, 2007, p. 371). Sleeter (2017), for example, argued that teacher education can “constructively confront” its privileging of “whiteness” through measures such as ensuring all teacher educators “situat[e] themselves within … an analysis of race” (p. 166) and “broaden the range of voices at the [teacher education] table” (p. 164). Similarly, Li et al. (2019) called for antiracist pedagogies that can foster new dispositions in teacher education. They add that in the case of preparing teacher candidates to teach English language learners (ELLs), alternatives to the “knowledge-transmission” (p. 87) model, which treats ELLs as a “side note” (p. 77), are needed. Moreover, de Jong and Naranjo (2019) asserted that knowledge for teaching ELLs could be embedded in teacher education through discussions of course readings. Tacit within these measures are questions about reading, such as Whose knowledge creates the texts in teacher education? RPEP undertook to explore such questions.

### Professional Learning

RPEP was built on the literature's identification that professional learning for teacher educators is important for promoting equity within teacher education (e.g., Naiditch et al., 2024) and our reading that this literature could be augmented through RPEP's posthuman orientation and focus on reading. There is a robust, pragmatic literature on design principles for professional learning for teacher educators that has been concentrated in Europe, with extensive scholarship coming from the Netherlands (e.g., Kelchtermans et al., 2018). Such principles are human-centric and include ensuring teacher educators have ownership over the content and process of their learning, setting professional learning within the real time of teacher educators’ teaching, offering teacher educators formal and informal learning opportunities that extend over time, creating teacher educator communities of practice, enlisting technologies as tools but not the focus of professional learning, and cultivating flexible pedagogies (Tack et al., 2021). More broadly, the literature stresses that teacher educators benefit from professional learning that is specifically intended for them (Berry, 2021), namely, that is relevant and responsive to their diverse professional and disciplinary contexts (Lunenberg et al., 2017). Decentering teacher educators to engage in professional learning that is attentive to reading and equity can highlight relational knowledge production.

## Methodology

The orientation of RPEP carried through into the study's speculative pedagogies of qualitative inquiry (Kuby & Christ, 2020) methodology. This posthuman methodology sees pedagogies as research. The term “pedagogies” here refers to the “method[s] and practice[s] of teaching” (p. 139) as well as “ways of know[ing] and be[coming] as” teachers “teach/learn/live” (p. 154). Pedagogies are multidimensional and include the practical, epistemological, and affective. In their complexity, pedagogies engage with learning in the face of the contingent, potentially surprising, and impartial nature of knowing; they are a lively experience of being “(in)query” (p. 138) with others. Pedagogies as a form of inquiry are also speculative: They are “an art of noticing” (p. 137) with an aim of producing “new understandings, questions, and insights” (p. 31). The collective of researchers and participants who engaged in RPEP were invited to “envisage futures” that “inform … what matters now” (Ross, 2023, p. 59), while recognizing “the complex knowledge-production spaces of learning and education” (p. 57), including what “we have inherited and what debates define what can and cannot currently be thought about or imagined” (p. 59). RPEP created the pedagogical conditions for teacher educators to learn with and through reading and equity while exploring the following questions: What is reading and equity in teacher education, and what is their relationship? What were the entanglements in the professional learning that produced this knowledge?

## Methods

RPEP enmeshed program development, research, and knowledge mobilization. It included recruited research participants and RPEP researchers. Together, this group represented pre-K-to-12 education and teacher education across all subjects.

### Participants

Recruited research participants were instructors from one initial teacher education program at one university in Ontario, Canada. The program was a two-year consecutive program (i.e., it required a first degree for entry), was divided into elementary and secondary teacher accreditation, and served approximately 800 teacher candidates. RPEP participant inclusion criteria were that participants had instructed at least one course in the initial teacher education program during the previous year or anticipated teaching at least one course in the upcoming year. Nine educators agreed to participate (Table 1). All were part-time instructors and held a minimum of a master's degree and professional experience.

**Table 1.** Participant Characteristics.

Participant pseudonym	Appointment status in the preservice program	Focus of preservice teaching	Professional background	Academic background
Celina	Part-time instructor	Early childhood education	Early childhood educator	PhD candidate (early childhood education)
Kim	Part-time instructor	Early childhood education	Early childhood educator	PhD candidate (early childhood education and science)
Ana	Part-time instructor	Content area literacies	Teacher and instructional designer	PhD (literacy education)
Pamela	Part-time instructor	Elementary language arts	Teacher	Master's (education)
Claudia	Part-time instructor	Content area literacies	Instructional designer and curriculum developer	PhD (educational sciences and technology)
Stuart	Part-time instructor	Elementary social studies	Teacher and school principal	Master's (education)
Martha	Part-time instructor	Special education and elementary language arts	Teacher	PhD (literacy education and assessment)
Amy	Part-time instructor	Education equity	Teacher and school administrator	PhD (education and equity studies)
Elaine	Part-time instructor	Elementary science	Teacher	Master's (science education)

In addition to the recruited participants, 10 research team members were co-learners in the professional learning. Of these, seven were faculty from four universities across Canada. Another team member was the Indigenous cultural educator, and two members were PhD students in education, one serving as a research assistant and one as the atelierista.

### Professional Learning

The group engaged in RPEP's 12-module online professional learning program. Following the speculative pedagogies’ notion that how one teaches “impacts what the academy is and becomes as well as impacts the communities” one researches with (Kuby & Christ, 2020, p. 12), the team sought to model pedagogical opportunities for generative reading engagements while exploring reading and equity. The modules were developed by the team on an online learning management system, then updated throughout implementation to respond to what was emerging. Each module focused on its own topic derived from the team's reading of the literature and allowed the group to “plug in” (Jackson & Mazzei, 2013) different ideas and texts into the question of reading and equity: (a) introduction to the project, team, and participants; (b) introduction to academic reading; (c) thinking with context; (d) thinking with teacher candidates; (e) thinking with cultural and linguistic diversity and reading; (f) thinking with texts, media, modalities, and disciplinary genres; (g) thinking with text procurement and authorship; (h) thinking with course delivery models and text forms; (i) thinking with before-reading pedagogies; (j) thinking with during-reading pedagogies; (k) thinking with post-reading pedagogies; and (l) preparation for symposium.

The modules were similarly structured and contained an introduction to the team member guiding the module, orienting questions, shared texts, and an invitation for a reading engagement (i.e., an opportunity to think with the module through a multimodal activity modeled by the atelierista). For example, for Module 5, an orienting question was: What does it mean to think with academic reading, Englishes, and multilingual repertoires? The texts to think with were a video on translanguaging (MuDiLe, 2017) and a multimedia literature review on second-language academic reading created by the guide. The engagement was inspired by Graff and Birkenstein's (2021) *They Say / I Say* and invited the group members to view the texts, select quotes, and record the quotes to form “They Say.” Beside the quotes, the members could record reactions or questions to form “I Say.” Then they could consider what was created through the juxtaposition.

Each module offered an online forum for sharing engagements and asynchronous conversation and a synchronous Zoom meeting opened by the cultural educator. The program also established a Padlet (Wallwisher, 2022) where the group could share resources and culminated in a public symposium.

### Data Generation

Data were generated before, during, and after the professional learning. Data generated before and during the program informed upcoming modules, and all data were used to explore the research questions.

Before the program, data were generated through research team-developed semi-structured interviews with the participants and their course syllabi. These interviews generated data concerning participants’ contexts (e.g., courses they taught), academic and professional preparation to teach in teacher education, and available supports for university teaching. Other questions asked about reading within the participants’ courses (e.g., How did the readings come to be on your course syllabus?), conceptions of reading (e.g., Tell us about a time a student read in a way that you thought was successful), understandings of reading and equity (e.g., What does this story say about reading and equity?), and their aspirations for RPEP.

Data generated during the program were the asynchronous online module discussions, recordings, and researcher field notes of synchronous Zoom meetings, and engagement artifacts.

After the program, data were generated through team-developed follow-up semi-structured interviews that featured engagement artifacts. As co-learners, the researchers also interviewed each other using the same protocol. The interviews considered future practices with reading (e.g., What new insights might you have about the demands readings place on students? How might your course now help students meet these demands?), conceptions of reading and reading pedagogies (e.g., What do you now understand to be the link between academic reading and equity, and how might you support a positive link?), the origins of insights (e.g., How were these understandings derived? Are there examples from the engagements to illustrate?), participation in the learning (e.g., How did you feel working with an atelierista?), and views on the design of RPEP.

### Analysis

Speculative pedagogies of qualitative inquiries conduct inquiry with theory (Kuby & Christ, 2020); hence, a thinking-with-theory approach (Jackson & Mazzei, 2013) was used for data analysis. Thinking with theory consisted of “plugging in” (Jackson & Mazzei, 2013, p. 262) one text or theory into another in an iterative process while generating and analyzing data as a team. This process involved trying to retain the complexities and situatedness of the phenomena in question and sharing with readers the theories that helped to shape readings of the data and the processes for composing with them.

The theories that the group was thinking with before, during, and (for researchers) after the program were entanglement (Barad, 2007), diffractive reading (Barad, 2007), and enacted agency (Wargo, 2018). Entanglement foregrounded the relational ontology (Bozalek & Zembylas, 2017) of how humans, matter, time, and space were brought together with/in the research and our considerations of reading and equity. We thought about what came together, how, and with what effects. Diffractive reading provided opportunities to bend our readings of data, texts, and other constituents of the program through each other. We diffracted data by returning to them through the group members’ and atelierista's engagements during the program and in the post-programmatic interviews. This reading allowed the destabilization of binaries (e.g., equitable/inequitable) and highlighted newness as constituents continuously (re)assembled to produce insights into reading and equity (Wargo, 2018). Shifting agency from the singular to the collective, enacted agency enabled the learning of the group to be greater than the sum of its parts.

Entanglement, diffractive reading, and enacted agency also foreground that the processes, practices, and effects of RPEP were particular: They were what they were because of who and what came together in circumstances that cannot be replicated. The study was “non-representational, not aspiring to represent a part of the reality that existed independently of the researcher[s] before the research” (Østern et al., 2023). Such research is incompatible with positivist methods that center on rigor (Kuby & Christ, 2020). Instead, the study sought a version of trustworthiness where research performs its value-laden truth through speculation and an articulation of its situatedness (Østern et al., 2023).

### Trustworthiness

The findings reflect that research like RPEP “asks” scholars “to trust that something unimaginable might come out that might change the world bit by bit” (Kuby & Christ, 2020, p. 135). The study considered the relationality between reading and equity and enabled speculative possibilities with theory to provide new awareness of reading in higher education and the future creation of inventive pedagogies toward equity. Learning together in RPEP allowed “a space to be uncertain, but in a ‘good’ way—meaning understanding wasn’t the (sole) goal but rather the goal was thinking, mucking, expanding, changing” (Kuby & Christ, 2020, p. 33), and seeing what could come, including what equity could become. Making and sharing these productions included measures that have been used to ensure trustworthiness, such as detailed descriptions of pedagogy, diffracting understandings back to and through the group, and all research team members being invited to coauthor this article. Those who opted in reviewed data, outlines, drafts, and revisions and engaged in conversation to find resonance in what was being shared and how. Furthermore, we followed additional plugging-in maneuvers from Jackson and Mazzei (2013). First, we attempted to disrupt the theory/practice binary through our design of the programmatic/study structure of RPEP. Second, we were “deliberate and transparent in what analytical questions” (p. 264) were made possible through our orientation. These questions included those related to the research concepts, namely, reading and equity, which in traditional research would have come with operational definitions, but in RPEP were open as questions. Last, the analysis involved multiple team members repeatedly going over the data, plugging it in and seeing plugging in as “a production of the new: the assemblage in formation” (Jackson & Mazzei, 2023, p. 2).

### Learnings

The project produced responses to the questions of reading and equity in teacher education and the entanglements in the professional learning that helped produce these responses. By reading data diffractively, that is, in relation to each other, and with sensitivity to who and what came together in productive entanglements, we identified relational learning that provoked new appreciations of reading and equity. Coproducing these appreciations were a plethora of sociomaterial (im)materialities, including modalities and knowledge. Navigating our learning required guides and a willingness to let go of traditional notions of academic reading and texts and narrow notions of equity.

### Critical Readings

The nature of enacted agency means that realizing links between reading and equity requires more than an individual teacher educator's awareness. This premise held true in the study. We found that the co-learning occurred through entangled (im)materialities that RPEP assembled. These (im)materialities included the existence of the study itself, opportunities for conversation among teacher educators, and multimodal conversations with the constituents of reading, such as texts and domains. Each of these conversations created new texts that could be diffracted into additional insights and speculations.

The existence of the study created sensitivity to reading and equity. For example, the pre-programmatic interview revealed the desire of participant Amy (all participant names are pseudonyms) to explore what had previously been tacit for her. When asked about her conception of the link between equity and reading, Amy said, “I can’t believe as someone who teaches in the field of equity that I never thought about the implications or the connections between reading pedagogies and equity…. I just saw … readings as the thing that you did.” Amy's comment hints at readerly (im)materialities and missing pedagogical opportunities when reading is just something “you d[o].” The research team expressed familiarity with Amy's recognition of the invisibility of reading in higher education; RPEP, for example, was born when co-author, Rachel, questioned her own university teaching, turned to colleagues and the literature to see what thought existed about reading and equity, and found an opening.

Thinking with others, beginning in the pre-programmatic interview, also produced insights about reading and equity, including connections that could inform pedagogies. Each realization begot another. For example, in continuing to reflect on why reading and equity had been invisible to her despite her expertise in equity studies, Amy pointed to the context of teacher education:

I keep asking myself … how much of [the ability to see equity and reading] is because of our disciplinary backgrounds?… If I [had] a literacy background would … this blind spot of mine be a little bit smaller … or is it the context of higher education where it's really easy … for that blind spot to be there, because the narrative is our students, they’re in this space because they’re great readers … because they’re really good at university? You just don’t really think that it's something that you need to think or talk about.

Later in this interview, Amy looked beyond reading and equity being just about teacher candidates’ facility with print: She discussed the importance of the “power” of “whose stories are told” and “whose stories aren’t told,” a point echoed by Ana in her pre-programmatic interview when responding to the question of reading and equity: “I think about who gets published and who doesn’t…. I have been grappling with … decolonization and having multiple voices and diverse voices.” Ana and Amy signaled future group insights through the exploration of the various (im)materialities whose entanglements beget (in)equities.

The pre-programmatic interview also produced questions about equity as it related to discourse and genre, with Pamela, for instance, remarking,

Academics are the pits at writing in terms of being aware of their audience, so the best textbooks really have in their mind's eye who the reader is and that that teacher reader is always looking for what does it look like in practice.

Pamela then expressed that equity looked like teacher educators being translators to bridge the gap between academic and professional discourses and domains, and she voiced a preference for education scholars to communicate in ways that she saw as commensurate with the discourse of teachers. She thus raised textual and readerly (im)materialities.

Early in the program, Elaine speculated about what reading could do for and to teachers, pointing to how reading might expose them, and ostensibly teacher candidates, to epistemological diversity. She said,

There's no way a teacher can experience all the different classrooms, all the different cultures…. [To] experience all the diversity we have in our world it's just impossible … but if you are engaged in … reading … you can get … a chance to understand different perspective[s] [and] different experience[s].

The program to come created opportunities to pose and ponder questions such as how reading in teacher education might (re)inforce binaries (e.g., practice and theory), generate professional norms (e.g., this is what teachers read), and how difference might be lived and read—queries that were important to conceptualizing readerly and other (im)materialities.

### Domains

When reading the data for what entanglements enacted agency and with what effects, we saw the program's pedagogies and materials co-producing insights about reading and equity. Following the pre-programmatic interviews, the group began to formally explore together through the program. RPEP was designed to create openings to collectively think with reading and equity in teacher education through a diversity of (im)materialities, including modalities (print, image), media (video, software), knowledge (academic, professional, Indigenous, artistic), spaces (Zoom, our classrooms), and domains (subject-specific disciplines, regulatory bodies).

The module organization was part of the entanglements that netted speculative effects. As described, modules contained invitations for engagements. Engagement artifacts were part of the diffractive nature of the pedagogies: They created new views of the orienting questions and texts. Module 2, for instance, an introduction to academic reading, invited participants to link a focal article with one of their own syllabi to embed professional learning and facilitate text-to-world, text-to-self, and text-to-text connections (Mantei & Fahy, 2018), a form of plugging in. The engagement was a riff on a content area reading strategy, the triple entry diary (Herman & Wardrip, 2012), which participants could adapt for use in their teaching.

**Figure 1.**
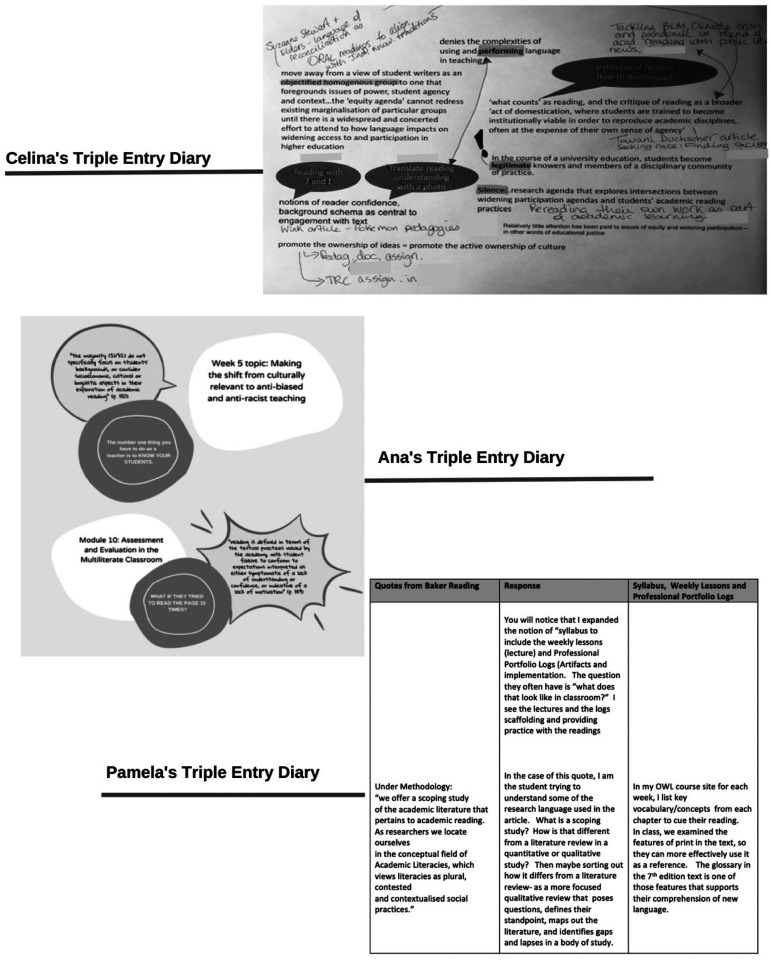
Triple entry diaries.

The artifacts from this engagement, such as those from participants Celina, Pamela, and Ana (Figure 1), illustrate how RPEP-assembled (im)materialities produced learning opportunities. Specifically, the artifacts demonstrate diffractive readings that plugged together the shared text (i.e., Baker et al., 2019), syllabi, and other texts and experiences with and through modalities, media, and textual features (e.g., layouts and color). They also reveal the movement of ideas across genres and domains. Celina's artifact, for instance, highlights quotations from the text through dark speech bubbles and orange highlights, asking questions of them in relation to her syllabi. Included is the statement “Notions of reader confidence, background schema as central to engagement with text,” with two bubbles connected to her syllabi saying “Reading with and !” and “Translate reading understanding with a photo.” Celina's artifact connected concepts from the text with pedagogies she was experimenting with in her classroom, while also making links with readings from her courses, such as the *Final Report of the Truth and Reconciliation Commission of Canada* (Truth and Reconciliation Commission of Canada, 2015). (The Truth and Reconciliation Commission, or TRC, was established in 2007 to facilitate reconciliation “among former students [of residential schools for Indigenous children], their families, their communities and all Canadians” [Indigenous Languages Act, Canada, 2019]. The TRC has published numerous reports, including *Calls to Action* [TRC, 2015], which identifies steps for teacher educators to support teacher candidates in developing and implementing curricula about residential schools in Canada.) Celina's artifact is a riot of annotation showing associations between readings and ideas, with exclamation marks noting key ideas and handwritten notes for and about practice. The artifact vibrates with ideas on reading and equity in teacher education coming together with Celina's courses, RPEP, and the readings.

Ana's artifact similarly signals a plugging in among ideas, though in ways that are different from the others. Her artifact enlists shapes and colors to set the text's quotations apart from content concerning her literacy course for secondary school content-area teachers (syllabi and readings). Ana offered a legend for reading the artifact, indicating that the pink (or light) bubbles contain quotes from the text; the white, pieces from her course syllabus; and the blue (or dark), “a thought or question I had.” Ana's artifact focuses on readerly (im)materialities in relation to text: It wonders of her teacher candidates, “What if they tried to read the page 10 times?” alongside a quote about the problem of the responsibility of academic reading failure being ascribed to students rather than to other (im)materialities or the intra-relation between (im)materialities.

Pamela's artifact is structured as a table that juxtaposes quotations from the shared text, her reading of the quotations, and reflections on her teaching of an elementary language arts course. This tripartite design shows how Pamela engaged with RPEP concerns and the text and documents Pamela's struggles reading the text. Through her struggles, Pamela expressed a sensitivity to what her students might feel and need with their course readings. Thus, the table's third column consists of pedagogical speculations to “cue” the “reading” of the teacher candidates. Pamela's artifact shows a visually and conceptually systematic attempt to address equity and reading in her teaching.

The Zoom module meetings created additional opportunities for seeing the entanglements of reading and equity. The presence of the cultural educator was central to these meetings. She introduced an explicit reckoning with power and privilege while juxtaposing semiotic and epistemological diversity as she reminded the group that the land we were on and its “original people” dictated our responsibilities as teacher educators, including our roles in promoting critical reading. In Module 2, for example, the cultural educator shared a key learning from RPEP, the forming of relationships:

The bottom line of the truth is to create … relationships, [you have] to be able to sit there and learn with your heart, mind, body, and spirit … to educate yourselves…. Most of the educational system is about, You read this book … and tell us what you know about it. Well, that's nice and wonderful, but if you’re not feeling it, and if you’re not seeing it, and you’re not creating relationships with the original people, then it really doesn’t mean too much…. There needs to be more education by the original people to the educators.

In examples like this, the cultural educator challenged the practice of reading an assigned book as the main route to the knowledge required to be a teacher. The cultural educator taught that reading pedagogies of equity was not about a mind extracting a message from a print text and teachers reinforcing this practice. Reading pedagogies that promote equity are instead radically relational, ethical undertakings that disrupt traditional academic reading and reorganize who and what is learning.

The cultural educator's juxtaposition of epistemologies and knowledge sources also converged with conversations about the need to relinquish the primacy of print and highlighted oral language and learning from Indigenous people by being with them, ideally on the land. Responding to a participant asking where they could learn more about an Indigenous view of child development, the cultural educator replied, “You’ll have to find somebody…. It's not written down … not everything is written down. It's about you sit and you learn that. It took me seven days to learn the seven stages of life; it's not something that is done one time.” The knowledge of child development that was, in traditional forms of education scholarship, codified in developmental psychology textbooks here became a living knowledge that was best acquired by listening intensively and engaging with more than one's mind. How to expand reading in text selections for teacher education was returned to in the module on text procurement and authorship. This module included a model of decolonizing knowledge sources from a library and information science program for preservice librarians that recruited Indigenous ways of knowing and knowledge sources (Hill et al., 2021).

**Figure 2.**
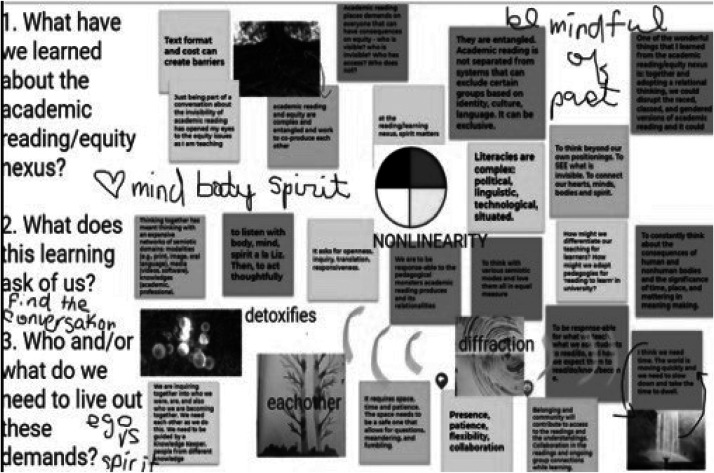
Jamboard.

RPEP learnings suggest the productive nature of pedagogically engaging a variety of media to help instantiate the collective and intra-relational nature of learning and unmooring sedimented academic knowledge. Artifacts from the Module 11 meeting, for instance, illustrate some of what RPEP was producing, how, and the threads of knowledge across the invitations of the program. Module 11 was a time of consolidation. The guide used a Jamboard (Jamboard, 2017), a collaborative online board, to invite everyone to share their responses to culminating RPEP questions. One cluster of these questions was, What have we learned about the academic reading/equity nexus? What does this learning ask of us? Who and/or what do we need to live out these demands? Figure 2 shows an image of this Jamboard, where the group added, annotated, and organized messages, creating a collective text. These are some examples of what the group said they learned: “Text format and cost [of textbooks] can create barriers,” “Just being part of a conversation about the invisibility of academic reading has opened my eyes to the equity issues as I am teaching,” and “They are entangled. Academic reading is not separated from systems that can exclude certain groups based on identity, culture[,] language.” As to the questions of what this learning was demanding of teacher educators and what teacher educators needed, the entries reverberate with the language of RPEP as everyone involved lived and made it together: “At the reading/learning nexus, spirit matters” (written above an image of the medicine wheel), and “One of the wonderful things that I learned from the academic reading/equity nexus is: together and adopting a relational thinking, we could disrupt the raced, classed, and gendered versions of academic reading.” Underscoring the linked nature of RPEP's exploration of equity and clearing the harm of reading, someone listed “each other” diagonal to an image of mycelium and the word “detoxifies.” According to the group, learning together with different knowledge and texts is a necessary condition of reading pedagogies of equity.

### Guides

An outcome of RPEP was a demand for the group to shift from being teacher educators to being teacher learners. This learning was exhilarating, as the vibrancy of the figures in this article suggests, but it was also challenging to leave the familiarity of old modes of thought and affect. RPEP created conditions for everyone and everything in the project to provide guidance to each other, with special guidance from the cultural educator and atelierista.

The cultural educator was a fundamental guide whose presence was integral to making visible what reading in higher education and its uses have taken for granted. As suggested in the previous section, the cultural educator was also a guide for how teacher educators could assume their responsibilities in reading and pedagogy on this land. Thinking with a cultural educator and embedding professional learning in the group's teaching meant that the Zoom meetings were a time to tell stories of practice and diffract them through the cultural educator's teachings. This rhythm was unexpected for some but valued by all. At the end of a meeting early in the program, for instance, Elaine stayed behind to ask the module's guide if she had participated in the module “correctly.” She expressed expectations of how the gatherings would go, anticipating highly structured sessions where people took turns posing and answering questions about the readings. The guide expressed that there was no wrong way to participate: “This is for you, it's your professional learning.” Elaine responded, “Awesome, okay. ‘Cause … [the cultural educator's] talk was like so much better, and I’m glad I was here for it.” The cultural educator guided the group through different ways of thinking with reading and equity in a search to live well together.

The group was also guided through the learning by the atelierista, who called on the visual to diffractively interrupt ossified text forms. The RPEP syllabus is a case in point. Instead of a flat, step-by-step didactic scope and sequence of topics where the syllabus was laid out as a table with columns and rows, as is typical for syllabi, the atelierista drew the syllabus as a mycelial network. The drawing was inspired by a team conversation about curriculum and the potential of the metaphor of mycelium to guide us (brown, 2017). The syllabus suggested the intra-relations of the project and attended to aesthetics, destabilizing the sterility of typical university syllabi. This metaphor returned repeatedly throughout RPEP, such as in engagement artifacts and the Jamboard entry about detoxifying, which is a function of mycelia.

The atelierista also modeled responses to the engagements, opening multimodal possibilities and the new thinking and feeling they could engender in contemplating reading and equity. Witness, for instance, the atelierista's engagement artifact for Module 3. This module invited participants to think with context and posed questions such as What does it mean to read in teacher education in a settler-colonial context? The shared texts for the module included the Indigenous Languages Act (2019) and a policy text governing teacher education (Ontario College of Teachers, 2017). The engagement invited participants to think with the (im)materialities that influenced their reading of policies. For her artifact, the atelierista, a teacher who had worked in a public library system, created a drawing and shared it in the forum.

I created a free-form shape, perhaps a shifting map, as we are thinking with lands and text.… My experience working for the public library system has been influencing my thinking about my own and the public's interaction with text…. Public acquisition policies help guide library purchases and reduce bias in selection of these purchases. I’ve been thinking about how the TRC documents need to be closely in conversation with such policies.

Thinking with the atelierista's artifact prompted comments in the forum from others, such as Claudia, who said, “I admire those of you who think in such a visual way … with a conciseness and accessible format.” A team member similarly stated, “I was quite drawn into [the] drawing. It really captured a different sense of relation when the ‘motion’ of relations is captured. The best I could do was arrows.” The atelierista's critical reading created opportunities to go beyond arrows, and its effects were also demonstrated in the artifacts we shared from Celina, Ana, and Pamela.

The contributions of the atelierista were additionally connected to thinking with the cultural educator and a variety of (im)materialities. For example, the atelierista shared photographs in the forum, saying:

In thinking further with Module 3, literacies, and policy documents, as well as our times in conversation with [the cultural educator], I was surprised to find a couple of days after our session that the eagle that sits outside my door … was surrounded by … turkey tail mushroom. This provoked me to further explore my backyard … to see what I could find via photography…. Today while looking again at the photos, I am curious to see the link in shape and structure of the turkey tail to the freestyle circle of influence I shared in the previous post…. As I work with these images, the TRC comes to mind, and the following calls to action are reflecting back to me through this engagement.

The atelierista next quoted from the TRC's (2015) calls for “Developing culturally appropriate curricula” and “Protecting the right to Aboriginal languages” (p. 2). Note that Celina's triple-entry diary also cited the TRC, suggesting how the engagements promoted critical thinking with intertextuality and the cultural educator.

## Discussion and Conclusions

Lacking robust treatment, reading, a lynchpin of higher education, including teacher education, has been an invisible impediment to equity (Baker et al., 2019), but RPEP is learning that it can be different. Theorizing of reading that is expansive in its understanding of what reading is and what it produces is necessary for creating higher education that can reckon with living well together in the Anthropocene (e.g., Burriss & Leander, 2024). Such theorizing is urgently needed to move the conversation on reading beyond a technical, skills orientation, which the literature has argued has been limiting and even harmful (Baker et al., 2019). Oriented through critical posthumanism and speculative pedagogies of qualitative inquiry, RPEP explored the reading/equity nexus, bringing visibility to it, and speculating on opportunities for critical reading. The inquiry recognized that its professional learning produced appreciations of reading and equity, potential pedagogies for critical reading, and knowledge concerning how these effects were coproduced. Each of these findings matters for the encouragement of a situated equity of reading in and through teacher education.

RPEP conceptualized reading as a sociomaterial, distributed practice produced through entangled (im)materialities that can (dis)able equity. Our reading of the literature suggested that these (im)materialities were textual, pedagogical, contextual, and readerly. Our study confirmed and elaborated on this literature by considering (im)materialities from the location of teacher education. The inquiry agreed that examples of (im)materialities that can make a difference to equity include those related to “disciplinary language and privileged forms of textual practice” (Baker et al., 2019, p. 145). Extending this finding are data such as Amy's interview about the nature of teacher education and the cultural educator's teachings about knowledge sources and text forms. These data saw textual (im)materialities as operating in relation to contextual ones, meaning the contact zones where texts, reading, and pedagogy become entangled with further (im)materialities such as the domain(s) of teacher education.

The findings also suggest that while the literature relays that readerly (im)materialities, those that focus on students, may be prominent opportunities for constraining equity by taking deficit views of readers (e.g., Baker et al., 2019), our inquiry highlighted pedagogical and textual (im)materialities to share the responsibility with readerly ones for coproducing readings of equity. The cultural educator, for instance, widened the gaze to the settler-colonial context in which reading was taking place and broadened the text forms and types that need to be included in teacher education. Pamela's interview also suggested the importance of the audience in writing and selecting texts, and the atelierista introduced the potential of multimodality to create new texts with which to think and pedagogies for increasing multimodal repertoires. These learnings mean that no longer can readers alone be understood as responsible for poor reading achievement.

Further confirming and extending the literature, the inquiry saw that reading and equity involved textual (im)materialities, such as modes, genres, and authorship, and pedagogical (im)materialities, such as how reading practices are embedded within learning opportunities (planned for or enacted). The findings, however, add to these (im)materialities the expectations of what reading is meant to accomplish, as in Elaine's contention that reading could be pedagogically framed as speculation. They also entwined textual, pedagogical, and contextual (im)materialities with knowledge systems (e.g., developmental psychology or Indigenous knowledge of child development) and what it means to be a teacher (i.e., a focus on knowledge in action called practice). Teacher educators can now attend to the (im)materialities from syllabi through to evaluation, asking questions such as those about the demands of texts, the types of domains and worlds they produce, and how pedagogies may be developed to promote critical readings.

The findings suggest that the inquiry's professional learning was a form of higher education reading that pedagogically leveraged (im)materialities to enact agency oriented toward criticality. Knowledge was cogenerated through entanglements that included the existence of the project, conversation to make the tacit visible, the uncovering of the taken-for-granted in teacher education, a consideration of the demands that reading places on teachers and candidates, the role of the teacher educator as a translator and guide into the domain of teacher education, the limits of discourse and modality, and the need to situate all reading entanglements within domains and their wider sociohistorical and material contexts (e.g., settler-colonialism). Also producing new understandings were the cultural educator and atelierista who guided learning with the oral and visual, enabling ideas to be diffracted back to the group, hence creating new texts and readings. Moreover, the materials of the professional learning allowed participants to unite across space and time to think with teaching, texts, and contexts. These findings underscore some of the sociomaterial elements in RPEP's pedagogical encounters and may guide professional learning for teacher educators and pedagogy more broadly.

## Supplemental Material

sj-docx-1-jlr-10.1177_1086296X251401121 - Supplemental material for Reading and Equity in Teacher Education: An Exploratory Study Supplemental material, sj-docx-1-jlr-10.1177_1086296X251401121 for Reading and Equity in Teacher Education: An Exploratory Study by Rachel Heydon, Lori McKee, Elizabeth Akiwenzie, Emma Cooper, Bronwyn Johns, Pamela J. McKenzie, Marianne McTavish, Sandra Poczobut, Carla Ruthes Coelho, Melody Viczko, and Zheng Zhang in Journal of Literacy Research

sj-docx-2-jlr-10.1177_1086296X251401121 - Supplemental material for Reading and Equity in Teacher Education: An Exploratory Study Supplemental material, sj-docx-2-jlr-10.1177_1086296X251401121 for Reading and Equity in Teacher Education: An Exploratory Study by Rachel Heydon, Lori McKee, Elizabeth Akiwenzie, Emma Cooper, Bronwyn Johns, Pamela J. McKenzie, Marianne McTavish, Sandra Poczobut, Carla Ruthes Coelho, Melody Viczko, and Zheng Zhang in Journal of Literacy Research

sj-docx-3-jlr-10.1177_1086296X251401121 - Supplemental material for Reading and Equity in Teacher Education: An Exploratory Study Supplemental material, sj-docx-3-jlr-10.1177_1086296X251401121 for Reading and Equity in Teacher Education: An Exploratory Study by Rachel Heydon, Lori McKee, Elizabeth Akiwenzie, Emma Cooper, Bronwyn Johns, Pamela J. McKenzie, Marianne McTavish, Sandra Poczobut, Carla Ruthes Coelho, Melody Viczko, and Zheng Zhang in Journal of Literacy Research

sj-docx-4-jlr-10.1177_1086296X251401121 - Supplemental material for Reading and Equity in Teacher Education: An Exploratory Study Supplemental material, sj-docx-4-jlr-10.1177_1086296X251401121 for Reading and Equity in Teacher Education: An Exploratory Study by Rachel Heydon, Lori McKee, Elizabeth Akiwenzie, Emma Cooper, Bronwyn Johns, Pamela J. McKenzie, Marianne McTavish, Sandra Poczobut, Carla Ruthes Coelho, Melody Viczko, and Zheng Zhang in Journal of Literacy Research

sj-docx-5-jlr-10.1177_1086296X251401121 - Supplemental material for Reading and Equity in Teacher Education: An Exploratory Study Supplemental material, sj-docx-5-jlr-10.1177_1086296X251401121 for Reading and Equity in Teacher Education: An Exploratory Study by Rachel Heydon, Lori McKee, Elizabeth Akiwenzie, Emma Cooper, Bronwyn Johns, Pamela J. McKenzie, Marianne McTavish, Sandra Poczobut, Carla Ruthes Coelho, Melody Viczko, and Zheng Zhang in Journal of Literacy Research
