# Supplementary material for: Reading and Equity in Teacher Education: An Exploratory Study
Source: J Lit Res. 2025 Dec 3;57(4):394–416. doi: 10.1177/1086296X251401121 (PMC12685152; doi:10.1177/1086296X251401121)
Supplement: sj-docx-1-jlr-10.1177_1086296X251401121 - Supplemental material for Reading and Equity in Teacher Education: An Exploratory Study [file sj-docx-1-jlr-10.1177_1086296X251401121.docx]

**مسودة رقم:** 0041.R4

**المؤلفون:** راشيل هايدون، لوري ماكي، إليزابيث أكيوينز، إيما كوبر، برونوين جونز، باميلا ج. ماكنزي، ماريان مكتافيش، ساندرا بوكزوبوت، كارلا روثيس كويلو، ميلودي فيتشكو، وزينج تشانج

**العنوان:** القراءة والمساواة في تعليم المعلمين: دراسة استكشافية

**مُوجَز**

تُعد القراءة ركنًا أساسيًا في التعليم العالي، إلا أنها تتطلب اهتمامًا تربويًا وبحثيًا متزايدًا. لقد شكّلت فجوات الممارسة والمعرفة تهديداتٍ لتحقيق المساواة، على الرغم من أن الطبيعة الدقيقة للعلاقة بين القراءة والمساواة في التعليم العالي غير معروفة. سعى مشروع "منهجيات القراءة والمساواة"، وهو برنامج تعليمي مهني ودراسة صُممت بالتعاون مع مُعِدّي المعلمين، إلى تكوين رؤى ثاقبة حول المساواة والقراءة في التعليم العالي. وقد استرشد فريق البحث بعلوم ما بعد الإنسانية النقدية والمناهج التربوية التأملية لمنهجية الاستقصاء النوعي، وقام بإنشاء بيانات مع تسعة مشاركين من مُعِدّي المعلمين. شملت مصادر البيانات منهجيات البرنامج التربوية، والمناقشات، والأدوات، بالإضافة إلى المقابلات التي أُجريت قبل البرنامج وبعده. حُلِّلت البيانات من خلال نهج التفكير من خلال النظرية، مع التركيز على التشابك، والقراءة العميقة، والوكالة المنفَّذة . حددت الدراسة نقاط التقاء نصية وسياقية وتربوية وقرائية في القراءة الأكاديمية تُمكّن/تُعيق فرص المساواة وعمليات إنتاج هذه المعرفة. وتُعدّ هذه النتائج هامة للمعلمين الراغبين في تعزيز المساواة في القراءة ومن خلالها.
