# Supplementary material for: Reading and Equity in Teacher Education: An Exploratory Study
Source: J Lit Res. 2025 Dec 3;57(4):394–416. doi: 10.1177/1086296X251401121 (PMC12685152; doi:10.1177/1086296X251401121)
Supplement: sj-docx-2-jlr-10.1177_1086296X251401121 - Supplemental material for Reading and Equity in Teacher Education: An Exploratory Study [file sj-docx-2-jlr-10.1177_1086296X251401121.docx]

**稿件编号：**0041.R4

**作者：**瑞秋·海登、洛里·麦基、伊丽莎白·阿基文齐、艾玛·库珀、布朗温·约翰斯、帕梅拉·J·麦肯齐、玛丽安·麦克塔维什、桑德拉·波佐布特、卡拉·鲁特斯·科埃略、梅洛迪·维茨科、郑章（音译）

**标题：**教师教育视域下阅读与公平的探索性研究

**摘要**

阅读是高等教育中处于核心地位，然而，在教学和研究层面都需要给予其更多关注。当前，实践层面与知识层面存在的差距，已然对教育公平构成了潜在威胁，但高等教育领域中阅读与公平之间关联的具体性质，至今仍未得到清晰明确的界定。“公平阅读教学法项目”是一项与教师教育者携手策划的专业学习项目与研究活动，其核心目标在于深入探究高等教育环境下的公平与阅读问题。本研究以批判性后人文主义和推测性定性探究教学法作为理论指引，研究团队与九位教师教育者参与者紧密合作，共同收集了丰富多元的数据。数据来源广泛，涵盖了项目所采用的教学法、参与者的讨论内容、项目产出的各类成果，以及项目开展前后的访谈记录。在数据分析环节，研究团队运用了“与理论共思”的方法，着重聚焦于要素交织、衍射式阅读和能动性等关键维度。通过深入分析，该研究成功识别出学术阅读中的文本、情境、教学法和读者等关键节点，这些节点在某种程度上促成（或阻碍）着公平机会的生成以及相关知识的构建过程。本研究成果对于那些期望在阅读实践过程中以及通过阅读这一途径来有效促进教育公平的教育者而言，具有至关重要的参考价值与启示意义。
