# Supplementary material for: Reading and Equity in Teacher Education: An Exploratory Study
Source: J Lit Res. 2025 Dec 3;57(4):394–416. doi: 10.1177/1086296X251401121 (PMC12685152; doi:10.1177/1086296X251401121)
Supplement: sj-docx-3-jlr-10.1177_1086296X251401121 - Supplemental material for Reading and Equity in Teacher Education: An Exploratory Study [file sj-docx-3-jlr-10.1177_1086296X251401121.docx]

**ID de manuscrit :** 0041.R4

**Auteurs :** Rachel Heydon, Lori McKee, Elizabeth Akiwenzie, Emma Cooper, Bronwyn Johns, Pamela J. McKenzie, Marianne McTavish, Sandra Poczobut, Carla Ruthes Coelho, Melody Viczko, et Zheng Zhang

**Titre :** Lecture et équité dans la formation des enseignants : Une étude exploratoire

**Résumé**

La lecture est centrale dans l'enseignement supérieur, mais elle nécessite une attention pédagogique et de recherche accrue. Les écarts dans les pratiques et les connaissances ont créé des menace pour l'équité, bien que la nature précise du lien entre lecture et équité dans l'enseignement supérieur reste inconnue. Le projet « Reading Pedagogies of Equity », un programme de développement professionnel et une étude conçue avec des formateurs d'enseignants, visait à produire des connaissances sur l'équité et la lecture dans l'enseignement supérieur. Ancré dans le posthumanisme critique et une méthodologie s'appuyant sur une 'pédagogie spéculative' de l'enquête qualitative, l'équipe de recherche a co-produit des données avec neuf formateurs d'enseignants participants. Les sources de données comprenaient les pédagogies mises en œuvre, les discussions et les artefacts issus du programme, ainsi que des entretiens menés avant et après le programme. Les données ont été analysées à l'aide d'une approche penser-avec-la-théorie axée sur l'intrication, la lecture diffractive et l'agentivité en acte. L'étude a identifié des nœuds textuels, contextuels, pédagogiques et liés au lecture dans la lecture académique qui rendent (im)possibles les opportunités d'équité et les processus de production de ces connaissances. Les résultats sont significatifs pour les éducateurs qui souhaitent promouvoir l'équité dans et par la lecture.
