# Supplementary material for: Reading and Equity in Teacher Education: An Exploratory Study
Source: J Lit Res. 2025 Dec 3;57(4):394–416. doi: 10.1177/1086296X251401121 (PMC12685152; doi:10.1177/1086296X251401121)
Supplement: sj-docx-4-jlr-10.1177_1086296X251401121 - Supplemental material for Reading and Equity in Teacher Education: An Exploratory Study [file sj-docx-4-jlr-10.1177_1086296X251401121.docx]

**Manuskript-Nummer:** 0041.R4

**Autoren:** Rachel Heydon, Lori McKee, Elizabeth Akiwenzie, Emma Cooper, Bronwyn Johns, Pamela J. McKenzie, Marianne McTavish, Sandra Poczobut, Carla Ruthes Coelho, Melody Viczko, & Zheng Zhang

**Titel:** Lesen und Chancengerechtigkeit in der Lehrerausbildung: Eine explorative Studie

**Zusammenfassung**

Lesen ist von zentraler Bedeutung in der Hochschulbildung, bedarf aber in Lehre und Forschung zunehmender Aufmerksamkeit. Wissens- und Praxisdefizite haben die Chancengleichheit beeinträchtigt, obwohl der genaue Zusammenhang zwischen Lesen und Chancengleichheit im Hochschulbereich weiterhin unklar ist. Das Projekt „Lesemethoden für Chancengleichheit“, ein in Zusammenarbeit mit Lehrenden entwickeltes Fortbildungsprogramm und eine Studie, zielt darauf ab, ein tieferes Verständnis von Chancengleichheit und Lesen im Hochschulbereich zu fördern.Ausgehend von kritischen Posthumanitäten und einer spekulativen Methodik der Pädagogik der qualitativen Forschung generierte das Forschungsteam Daten mit neun teilnehmenden Lehrerausbildern. Zu den Datenquellen gehörten die Pädagogik, Diskussionen und Artefakte des Programms sowie Befragungen vor und nach dem Programm. Die Daten wurden anhand eines theoretischen Ansatzes analysiert, der sich auf Verflechtungen, diffraktives Lesen und gelebte Handlungsfähigkeit konzentrierteDie Studie identifizierte die textuellen, kontextuellen, pädagogischen und leserbezogenen Verträge beim akademischen Lesen, die gleiche Chancen in den Möglichkeiten und Prozessen bieten, die zur Generierung dieses Wissens notwendig sind.Die Ergebnisse sind für Pädagogen von Bedeutung, die Chancengerechtigkeit im und durch das Lesen fördern möchten.
