# Supplementary material for: Reading and Equity in Teacher Education: An Exploratory Study
Source: J Lit Res. 2025 Dec 3;57(4):394–416. doi: 10.1177/1086296X251401121 (PMC12685152; doi:10.1177/1086296X251401121)
Supplement: sj-docx-5-jlr-10.1177_1086296X251401121 - Supplemental material for Reading and Equity in Teacher Education: An Exploratory Study [file sj-docx-5-jlr-10.1177_1086296X251401121.docx]

**ID del Manuscrito:** 0041.R4

**Autores:** Rachel Heydon, Lori McKee, Elizabeth Akiwenzie, Emma Cooper, Bronwyn Johns, Pamela J. McKenzie, Marianne McTavish, Sandra Poczobut, Carla Ruthes Coelho, Melody Viczko, & Zheng Zhang

**Título:** Lectura y Equidad en la Formación del Profesorado: Un Estudio Exploratorio

**Extracto**

La lectura es fundamental en la educación superior, pero requiere una mayor atención pedagógica y de investigación. Las lagunas en la práctica y en los conocimientos han creado amenazas para la equidad, aunque se desconoce la naturaleza exacta del nexo entre lectura y equidad en la enseñanza superior. El Proyecto de "Pedagogías de la Lectura para la Equidad", un programa de aprendizaje profesional y estudio diseñado con formadores de docentes, pretendía aportar ideas sobre la equidad y la lectura en la enseñanza superior. Orientado a través de las post-humanidades críticas, y una pedagogía especulativa de metodología de investigación cualitativa, el equipo de investigación generó datos con nueve participantes formadores de docentes. Las fuentes de datos incluyeron las pedagogías del programa, debates y artefactos, además de entrevistas previas y posteriores al programa. Los datos se analizaron mediante un enfoque teórico centrado en el entrelazamiento, la lectura difractiva, y la voluntad representada. El estudio identificó nodos textuales, contextuales, pedagógicos, y nodos en la lectura académica que disminuyen la posibilidad de oportunidades para la equidad, y los procesos para producir este conocimiento. Los resultados son significativos para los educadores que desean promover la equidad en, y a través de, la lectura.
